# Ezrin promotes breast cancer progression by modulating AKT signals

**DOI:** 10.1038/s41416-019-0383-z

**Published:** 2019-02-26

**Authors:** Nan Li, Jienan Kong, Zhenhua Lin, Yang Yang, Tiefeng Jin, Ming Xu, Jie Sun, Liyan Chen

**Affiliations:** 1 Department of Pathology and Cancer Research Center, Yanbian University Medical College, Yanji, 133002 China; 2 Department of Pathology, The First Affiliated Hospital of Dalian Medical University, Dalian, 116011 China; 3 Department of Biochemistry and molecular biology, Yanbian University Medical College, Yanji, 133002 China; 4 Key laboratory of the Science and Technology Department of Jilin Province, Yanji, 133002 China

**Keywords:** Biomarkers, Breast cancer, Breast cancer, Biomarkers

## Abstract

**Background:**

Ezrin, which is known as a cytoskeleton linker protein, is closely linked with the metastatic progression of cancer and is frequently abnormally expressed in aggressive cancer types. However, the possible involvement of Ezrin in metastasis and angiogenesis in breast cancer remains unclear.

**Methods:**

Immunohistochemical analysis of Ezrin was performed on both BC samples (*n* = 117) and normal epithelium samples (*n* = 47). In vivo and in vitro assays were performed to validate the effect of Ezrin on AKT pathway-mediated BC progression.

**Results:**

In this study, Ezrin was found to be upregulated in BC tissues, which was linked with aggressive tumour characteristics and poor prognosis. Moreover, we showed that Ezrin promotes BC proliferation, migration, invasion, and angiogenesis in vitro and in vivo. Mechanistic analysis showed that Ezrin interacted with AKT, and promoted its kinase activity, thereby regulating the AKT pathway in BC.

**Conclusions:**

In all, we propose a model for an Ezrin/AKT oncoprotein axis, which provides novel insight into how Ezrin contributes to BC progression.

## Background

Breast Cancer (BC) is a common malignancy and a significant cause of death in female worldwide.^1,2^ Every year, more than 1.3 million women are diagnosed with BC, and nearly 450,000 women die from it. Invasion and metastasis, which are estimated to be responsible for ~90% of all cancer-related deaths,^3,4^ are the primary factors that result in the failure of BC treatment and poor prognosis. Even in node-negative BC patients, 25% of patients develop metastasis.^5^ The 5-year survival rate is dramatically reduced in patients with distant metastasis. Very few stable biomarkers have been identified for risk evaluation or clinical outcome prediction in BC metastasis, although a considerable number of studies have been conducted. Hence, further investigations are necessary.

Previous studies have confirmed that metastasis is a complex process involving a series of changes, such as mesenchymal transition of local cancer cells, reorganisation of actin cytoskeleton, remodelling of the micro-environment and colonisation of metastatic cells.^6^ Among these changes, the process of mesenchymal transition in cancer cells, which defined as EMT, has attracted much attention in studies of BC metastasis.^7,8^ Clinical studies have revealed that EMT is closely associated with tumour metastasis and poor prognosis, and is considered the central mechanism responsible for metastasis in multiple cancer types.^9,10^ Therefore, inhibiting the EMT pathway of cancer cells as well as intervening with the key proteins in EMT-related pathways might provide insight into BC progression and greatly benefit our understanding of BC metastasis.

Angiogenesis also plays a major role in tumour growth, progression, and metastasis. As tumours progress, nutrients and oxygen become depleted within the core of the tumour, which induces the production of angiogenic growth factors.^11^ These growth factors bind to receptors on nearby quiescent endothelial cells (ECs) in pre-existing capillaries, leading to their activation and proliferation, eventually leading to the formation of new vessels.^12^ Blood vessels enable the exchange of nutrient and catabolites for cancer cells and allow communication between primary and metastatic tumours.^13,14^ Therefore, angiogenesis, the process of new blood vessel growth, is crucial for cancer development and is a potential target for cancer therapy.

Ezrin, an important member of the Ezrin-radixin-moesin (ERM) family of cytoskeleton-associated proteins, is a transit protein between membrane proteins and actin filaments.^15,16^ Nevertheless, emerging evidence has demonstrated that Ezrin may serve as a metastasis-related oncogene through modulating multiple cellular processes, including the formation of microvilli, maintenance of cellular morphology and intercellular connections, and promotion of cellular motility and invasion.^17–20^ Our group also reported that Ezrin was found to be overexpressed in cervical cancer, and its expression was closely related to metastasis and poor prognosis.^21^ Although multiple cancer-promoting activities of Ezrin have been described, the roles of Ezrin in metastasis and angiogenesis of BC remain largely unknown.

Our current study demonstrated that high expression of Ezrin indicated the high tumour invasion and poor prognosis in BC. Furthermore, functional experiments validated Ezrin as a positive regulator of EMT progression and angiogenesis in BC. Mechanistically, we demonstrated that Ezrin interacted with AKT and activated its downstream signalling, which eventually led to enhanced metastasis and angiogenesis in BC cells.

## Materials And Methods

### Reagents

Antibodies against Ezrin, E-cadherin, Zo-1, Vimentin, Snail, Slug, p-AKT, p-mTOR, p-S6, p-4EBP1, AKT, mTOR, S6, 4EBP1, CD34 and GAPDH were purchased from Cell Signaling Technology (Boston, USA). MMP9, HIF1α and VEGF were purchased from Santa Cruz (Dallas, USA).

### Clinical specimens

Three primary BC tissues with paired adjacent normal breast tissues were snap frozen in liquid nitrogen and tored at −80 °C until use. The histopathology of each specimen was reviewed on the hematoxylin and eosin-stained tissue section to confirm diagnosis and tumour content at least 70% of tumour cells in the tissue sample. The study of 117 paraffin embedded BC samples, as well as 47 normal epithelium samples were conducted. These samples were selected randomly from patients who underwent surgery between 2003 and 2008, with strict follow-up for survival status. Clinicopathological classification and staging were determined according to the American Joint Committee on Cancer (AJCC) criteria. Clinical information on the samples is summarised in Supplemental Table 1.

### Cell culture and transfection

The BC cell lines (MDA-MB-231 and MCF-7) were purchased from the ATCC. These cell lines were cultured in DMEM medium (Gibco, Gaithersburg, MD, USA) supplemented with 10% fetal calf serum, 2 mmol/L l-glutamine, and 100 U/ml penicillin/streptomycin in humidifies 5% CO_2_ at 37 °C.

We purchased three different Ezrin siRNA, including si-Ezrin-1, si-Ezrin-2 and si-Ezrin-3, from RIBOBIO (China). According to the KD effect, control siRNA (si-control), si-Ezrin-2 and si-Ezrin-3 were used in this study. The sequence of si-Ezrin-2 and si-Ezrin-3 were 5′-AAGGAAUCCUUAGCGAUGAGA-3′ and 5′-GGGCCAAGTTCTACCCTGAAG-3′. Cells were transfected with 30 nM siRNA using Lipofectamine 3000 (Invitrogen) according to the manufacturer’s instructions.

Human Ezrin cDNA was purchased form (You Biosciences, Hunan, China) and cloned into the pDONR223 plasmid. The Ezrin plasmid and corresponding empty vector were transfected into BC cells (MDA-MB-231 and MCF-7) using Lipofectamine 3000 reagent (Invitrogen) following the manufacturer’s protocol.

### Western blot

Whole-cell protein extracts were prepared with RIPA buffer containing a protease inhibitor mixture. Equal protein samples were separated on 10.5% SDS polyacrylamide gels and transferred to PVDF membranes (Immobilon P, Millipore, Bedford, MA). Membranes were blocked with 5% fat-free milk and probed with primary antibodies at 4 °C overnight, followed by probing with second antibody at RT for 2 h. Detection by enzyme-linked chemiluminescence (ECL) was performed according to the manufacturer’s protocol. Results were analysed quantitatively using Chemiluminescent and Fluorescent Imaging System. The detailed information was described previously.^21^

### Immunoprecipitation

BC cells were lysed with immunopreciptation (IP) lysate buffer (Beyotime, Shanghai, China) and the protein extracts were isolated. A volume of 25 μl Protein G-agarose beads/tube (Beyotime, Shanghai, China) were washed three times with buffer, followed by incubation with anti-Ezrin antibody, anti-AKT antibody or IgG antibody at 4 °C with rotation for 1 h. Incubated equal amounts of protein with beads at 4 °C overnight. Then the beads were resuspended in 3 × SDS Sample Buffer and boiled for 5 min. Western blotting analysis was performed with the standard protocol.

### Immunofluorescence

Cells grown in six-well culture slides fixed with 4% paraformaldehyde for 15 min, permeabilised with 0.5% Triton X-100 (CWBIO, China) and blocked with 3% BSA for 2 h. Cells were incubated with primary antibody in 3% BSA at 4 °C overnight, washed three times with PBS, incubated with Alexa Fluor 488 or Alexa Fluor 546-labelled secondary antibody (Invitrogen) in 3% BSA for 2 h, and then analysed by Leica SP5II confocal microscope.

### MTT assay

BC cells were seeded into 96-well plates at a concentration approximately 5000 cells per well, and then transfected with the corresponding compounds. Next, the MTT solution (100 μl) was added to each well of the plate at 0, 24, 48 and 72 h, and incubated for 4 h at 37 °C. Remove the medium from the well and add 100 μl DMSO into each well. The relative number of surviving cells was assessed by measureing the absorbance at 590 nm.

### Colony-formation assay

For colony-formation assays, cells were plated on 6-well plates at a concentration of 5000 cells per well and the medium was replaced every 3 days. Cells were fixed and stained with 1% crystal violet after incubating for 2 weeks.

### Wound healing assay

Cells were seeded in a 6-well plate and cultured for 24 h to form confluent monolayers. A wound was created by dragging a pipette tip through the monolayer, and plates were washed using pre-warmed PBS to remove cellular debris. Cell migration was monitored for 0 h and 12 h, and images were captured at each time point using a digital camera attached to an inverted phase contrast microscope.

### Cell migration and invasion assays

The migration and invasion abilities of cells were assessed using non-Matrigel-coated and Matrigel-coated Transwell inserts (BD Biosciences, San Diego, CA, USA), respectively. The assays and counting of migrating or invading cells were performed as described previously.^21^

### Vasculogenic mimicry analysis

The 96-well plates were coated with 60 μl Matrigel solution diluted as a 1:1 mixture of High Concentration Matrigel and culture medium without FBS, penicillin and streptomycin solution at 4 °C. The plate was allowed to polymerise for 4 h at 37 °C. MDA-MB-231 and MCF-7 cells, transfected with with si-Ezrin-2, si-Ezrin-3 and si-control in DMEM medium without FBS, were seeded at density of 3 × 10^4^ per well. After incubation at 37 °C for 4 h, the formation of capillary-like structures was captured under microscope.

### Matrigel tube formation assay

HUVECs were cultured at 37 °C in a 96-well plate coated with Matrigel (BD Biosciences, San Diego, CA, USA) diluted at 1:1 in cell culture medium at 4 °C. The plate was allowed to solidify for 4 h at 37 °C before cell seeding. The conditioned medium was collected from supernatant fluid of 48 h cultured MDA-MB-231 and MCF-7 cells with different transfections and filtered using 0.45 μm filter. HUVECs in 150 μl conditioned medium diluted at 2:1 in cell culture medium without FBS, were seeded at density of 2 × 10^4^ cells per well. After incubation at 37 °C for 4 h, the formation of capillary-like structures was captured under microscope.

### Chick chorioallantoic membrane assay (CAM assay)

To assess the effect of Ezrin on the pre-existing vasculature, we performed ex vivo CAM assay according to the previously described method with slight modifications.^21^ Ethics approval was obtained by the University of Yanbian Animal Ethics Committee. Embryonic eggs were incubated in humidified (65–70%) chamber at physiological temperature for about 7 days with their CAM exposed to visualise the angiogenesis. At 8th day, a total of 1 × 10^6^ MDA-MB-231 and MCF-7 cells transfected with si-control or si-Ezrin were added in sterile rubber rings on the exposed CAM and window-sealed eggs were incubated for additional 48 h. After incubation, the CAMs were photographed with a microscope (Olympus BX51) after fixation (methanol: acetone = 1:1).

### Animal studies

All procedures involving animals and their care in this study were approved and performed by the animal ethics committee of the Yanbian University, China. MDA-MB-231 cells (3 × 10^6^) transfected with si-control or si-Ezrin-3 was implanted subcutaneously in the left or right flank of 5-week-old BALB/c nude female mice (Vital Rivers, Beijing, China) to establish tumour model. Then the mean tumour weight was measured. For detecting lung metastases, BC cells were injected into the tail vein of nude mice. The lungs were collected, and the surface nodules were counted. H&E was performed for histological examination. Animals were sacrificed after 35 days of treatment, and a part of each tumour was fixed in formalin; another part of each tumour was chopped and immediately frozen in liquid nitrogen for further analyses.

### Immunohistochemistry (IHC)

IHC was processed in accordance with a previously described protocol.^21^ Tissue sections were deparaffinised, rehydrated and incubated with 3% H_2_O_2_ in methanol for 15 min at RT to eliminate endogenous peroxidase activity. Antigen retrieval was performed by placing the slides in 0.01 M sodium citrate buffer (pH 6.0) at 95 °C for 20 min. The slides were then incubated with the primary antibody at 4 °C overnight. After incubation with the secondary antibody at RT for 1 h, immunostaining was developed using DAB, and the slides were counterstained with hematoxylin.

Two pathologists who did not possess knowledge of the clinical data examined and scored all tissue specimens. In case of discrepancies, a final score was established by reassessment by both pathologists on a double-headed microscope. Briefly, the IHC staining for Ezrin was semi-quantitatively scored as ‘-’ (negative) (no or less than 5% positive cells), ‘ + ’ (5–25% positive cells), ‘ + + ’ (26-50% positive cells) and ‘ + + + ’ (more than 50% positive cells). The cytoplasmic expression pattern was considered as positive staining. Tissue sections scored as ‘ + + ’ and ‘ + + + ’ were considered as strong positives (high-level expression) of Ezrin protein.

### Statistical analysis

The data analysis was performed using SPSS 17.0 software and GraphPad Prism 6.0 software. Group comparisons for continuous data were done by t-test for independent means or one-way ANOVA. Survival curves were calculated using the Kaplan–Meier analysis. Biochemical experiments were performed in triplicate and a minimum of three independent experiments were evaluated. The value of *P* *<* 0.05 was considered statistically significant.

## Results

### Ezrin expression was upregulated in BC and correlated with a poor outcome

**Fig. 1:**
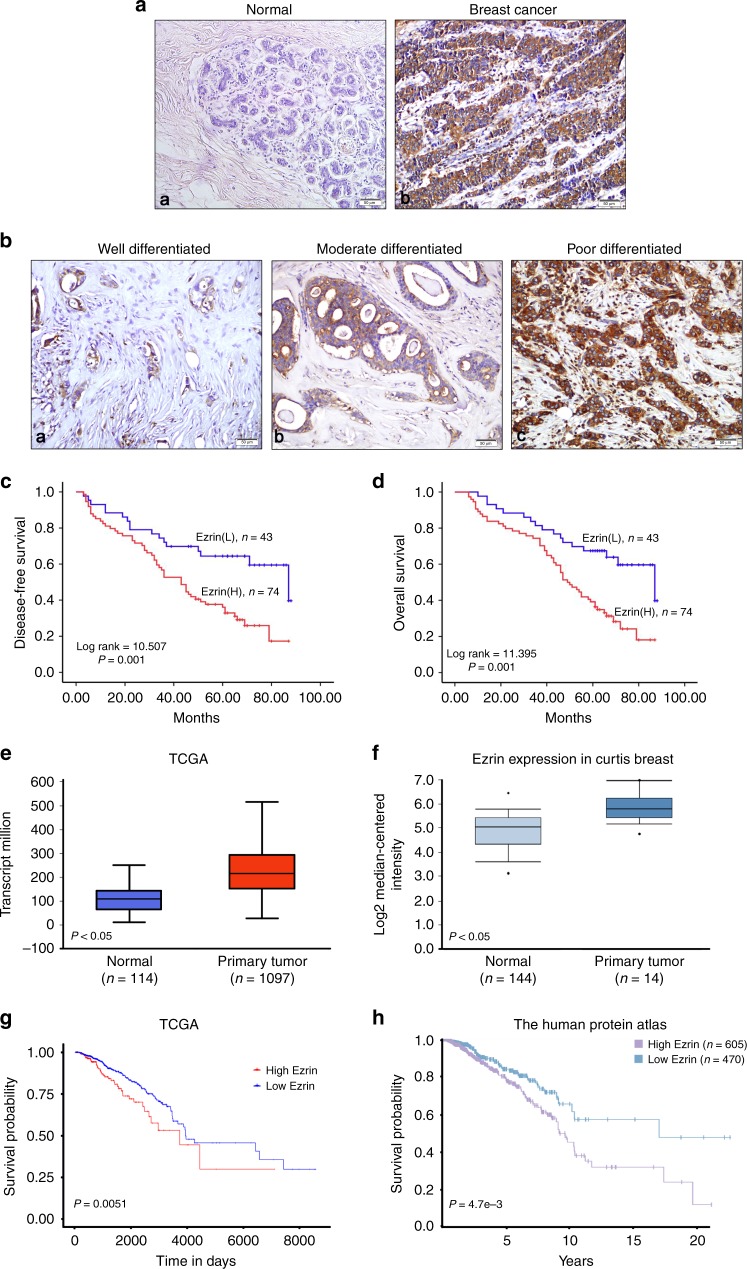
Ezrin expression is increased in BC and associated with poor outcome. **a** Ezrin expression in normal breast tissues (**a**) and BC tissues (**b**) was examined by IHC. Representative examples of Ezrin staining are shown. (**b**) Representative images of IHC staining for Ezrin in different grade BC tissues. **c**, **d** Kaplan–Meier survival analyses were conducted to evaluate the influence of Ezrin on patient disease-free survival (**c**) and overall survival (**d**). **e**, **f** Analysis of Ezrin expression in TCGA (**e**) and Oncomine (**f**) data set. **g**, **h** The implication of Ezrin in survival of patients with BC was determined in TCGA’s cohort (**g**) and HPA’s cohort (**h**)

To investigate the role of Ezrin in human BC, we first examined Ezrin expression in 117 pairs of BC and 47 normal breast tissues by IHC. The results revealed that Ezrin protein level was markedly upregulated in BC tissues (Fig. 1a, b). Here, 74 of 117 BC tissues (positive rate: 63.2%) and 15 of 47 normal tissues (positive rate: 31.9%) were found positive for Ezrin expression (*P* < 0.05). The clinicopathological analysis revealed that Ezrin expression was closely correlated with tumour differentiation (*P* = 0.015), late TNM stage (*P* = 0.004) and LN metastasis (*P* = 0.003) (Supplemental Table 1). Kaplan–Meier survival analysis showed that patients with high Ezrin expression had significantly shorter survival time (Fig. 1c, d).

We further analysis the mRNA expression of Ezrin in BC tissue samples from The Cancer Genome Atlas (TCGA), and observed that Ezrin levels were significantly upregulated in human BC tissues (Fig. 1e). In addition, Oncomine dataset confirmed that the expression level of Ezrin was significantly higher in BC than that in normal tissues (Fig. 1f). Further survival analysis using TCGA cohort and The Human Protein Atlas showed that high Ezrin group had obvious shorter survival time than those in low Ezrin group (Fig. 1g, h).

### Ezrin promotes cell proliferation and tumourigenesis of BC

**Fig. 2:**
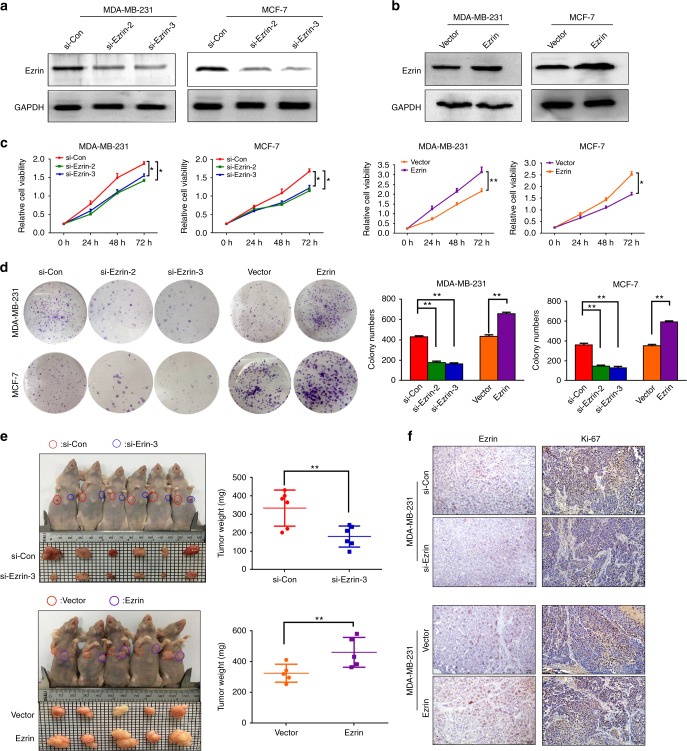
Ezrin regulates BC cell growth. **a**, **b** The protein expression of Ezrin in the constructed MDA-MB-231 and MCF-7 cells was confirmed by Western blot analysis. GAPDH was used as a loading control. **c**, **d** Cell proliferation was examined by MTT (**b**) and colony formation (**c**) in the constructed cells, and (**e**) Indicated that cells were injected into the nude mice. The tumours were dissected at day 35 and weighted. **f** Expression of Ezrin and Ki67 in the xenograft tumours tissues was detected by IHC

To determine the biological functions of Ezrin in BC progression, Ezrin expression was silenced by transfecting Ezrin-siRNA (si#1, si#2 and si#3) into MDA-MB-231 and MCF-7 cells (Supplemental Fig. 1). Si#2 and si#3 were selected to establish Ezrin-depleted (Ezrin-siRNA) cells, leading to low levels of Ezrin. We also established stable overexpression of Ezrin in MDA-MB-231 and MCF-7 cells at the same time. Western blot was performed to measure the protein levels of Ezrin expression (Fig. 2A, b). MTT and colony assays revealed that Ezrin knockdown markedly inhibited cell proliferation and clonogenicity, whereas Ezrin overexpression enhanced cell proliferation and clonogenicity (Fig. 2c, d).

We further investigated the effects of Ezrin on tumourigenesis using xenograft mouse model to verify the physiological relevance of our in vitro observations. As shown in Fig. 2e, the weights of tumours that generated from the Ezrin-depleted cells were markedly decreased in comparison with the si-con group. In contrast, the weights of tumours were significantly increased in Ezrin-overexpressed group compared to the control group. Moreover, IHC staining found that the Ezrin-depleted tumour tissues had reduced numbers of Ki67-positive cells, whereas the Ezrin overexpressed group had higher Ki67 proliferation indexes (Fig. 2f). Together, these results indicated that Ezrin plays an important role in the BC proliferation.

### Ezrin promotes BC metastasis via EMT in vitro and in vivo

**Fig. 3:**
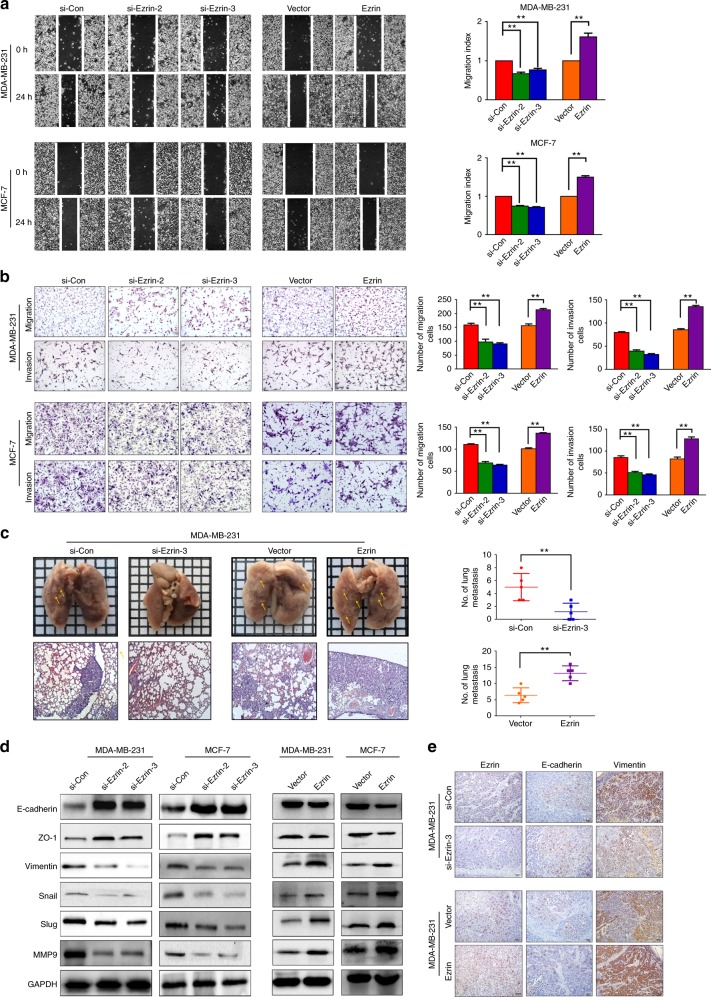
Ezrin promotes BC metastasis and invasion in vitro and in vivo. **a**, **b** Migration and invasion capacity of Ezrin in MDA-MB-231 and MCF-7 cells was examined by wound healing assay (**a**), transwell migration and invasion assay (**b**). **c** Indicated cells were injected into the mice through the tail vein. The metastatic nodules in the lungs were sectioned and counted. Representative micrographs of HE staining (lower panel) and the number of lung metastasis (right) were shown. **d** Western blot analysis of EMT markers in the constructed MDA-MB-231 and MCF-7 cells. GAPDH was used as the loading control. (**e**) Expression of E-cadherin and Vimentin in the xenograft tumours tissues was detected by IHC

Metastasis is responsible for poor outcome of BC.^22^ The clinical association study has found that overexpression of Ezrin was significantly associated with BC metastasis. To investigate the effects of Ezrin on the metastatic ability of BC cells, we performed wound-healing and Transwell assays. As shown in Fig. 3a, the wounds were healed more efficiently in the si-con BC cells than in the Ezrin-depleted cells. Knockdown of Ezrin also significantly reduced the ability of migration and invasion of BC cells (Fig. 3b). In contrast, the migration and invasion ability were evidently increased in Ezrin overexpressed cells compared to control cells (Fig. 3a, b). The in vivo metastatic experiments were applied to further validate the role of Ezrin in BC metastasis. For tail vein injection experiment, 6 weeks after injection, the metastatic nodules on the lung sections of tumour xenografts were observed, and we found that when compared with control group, the mice injected with Ezrin-depleted cells had fewer lung metastases and with Ezrin overexpressed cells had more of that (Fig. 3c). Histological photomicrographs of H&E stained lung tissue sections from each mouse were further confirmed the presence of lung metastases. Collectively, these data suggested an important role of Ezrin in BC metastasis and invasion.

Since EMT is an important component in tumour metastasis and invasion,^23^ we next investigate the effect of Ezrin on EMT in BC. Interestingly, we found that when Ezrin was silenced, the BC cells attained an epithelial morphology and lost their migratory capability, whereas Ezrin overexpressed cells acquired a dispersed, spindle-shaped morphology (Supplemental Fig. 2A). Using Western blotting and IF, we detected increased levels of epithelial cell markers (E-cadherin and ZO-1) and decreased levels of Vimentin, Snail, Slug and MMP9 in Ezrin-depleted cells (Fig. 3d and Supplemental Fig. 2B). Conversely, the opposite effect was found in Ezrin overexpressed cells (Fig. 3d). Consistent with the in vitro data shown above, the tissue sections from the subcutaneous tumour indicated that the expression of E-cadherin was increased and the expression of Vimentin was decreased in Ezrin-depleted cells compared with control group (Fig. 3e). The result was further confirmed in Ezrin overexpressed group. Overall, these results demonstrated that Ezrin is likely to promote BC cell migration and invasion via the induction of EMT.

### Ezrin promotes BC cells angiogenesis in vitro and in vivo

**Fig. 4:**
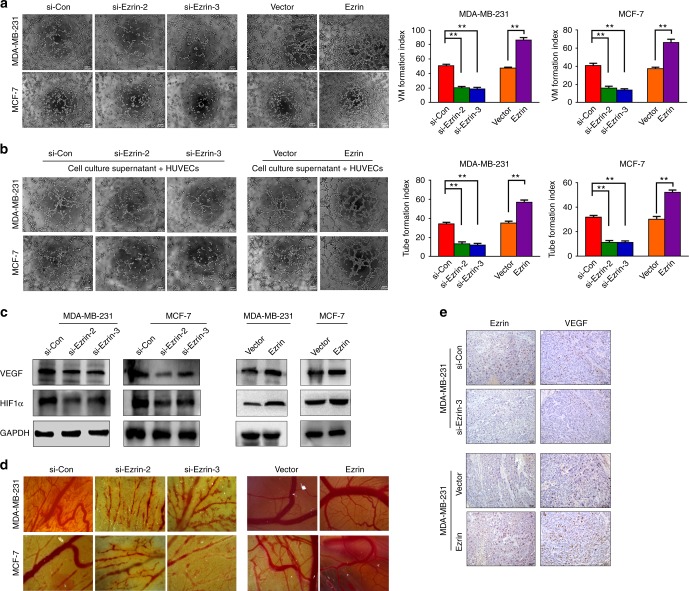
Ezrin promotes tumour angiogenesis in vitro and in vivo. **a**, **b** Vasculogenic mimicry assay (**a**) and tube formation assay (**b**) were performed in MDA-MB-231 and MCF-7 cells. **c** Western blot analysis of VEGF and HIF1α in the constructed MDA-MB-231 and MCF-7 cells. GAPDH was used as the loading control. **d** CAM assays were performed to confirm the effect of Ezrin on tumour angiogenesis ex vivo. **e** Expression of VEGF in the xenograft tumours tissues was detected by IHC

Given that angiogenesis is crucial for the metastasis and progression of cancer,^24,25^ we explored whether Ezrin has a role in BC angiogenesis. By performing vasculogenic mimicry and microtubule formation assay, we detected that the vascular mimicry and microtubule formation ability of HUVECs were reduced in the Ezrin depletion cells, but increased in the Ezrin overexpressed cells (Fig. 4a, b) (Supplemental Fig. 3). Western blot analysis was further performed and revealed that silencing of Ezrin reduced, and overexpression of Ezrin increased the expression levels of VEGF and HIF1α (Fig. 4c), suggesting that Ezrin may have pro-angiogenic properties in BC.

We also assessed the effect of Ezrin on ex vivo angiogenesis through CAM assay and found that the down-regulation of Ezrin decreased the angiogenic effects of BC cells. Conversely, up-regulation of Ezrin increased the angiogenic effects of BC cells (Fig. 4d). In accordance with this evidence, our results showed that the expression of VEGF was down-regulated or elevated in Ezrin-depleted or Ezrin overexpressed BC cells (Fig. 4e).

### Ezrin mediated BC progression by targeting AKT

**Fig. 5:**
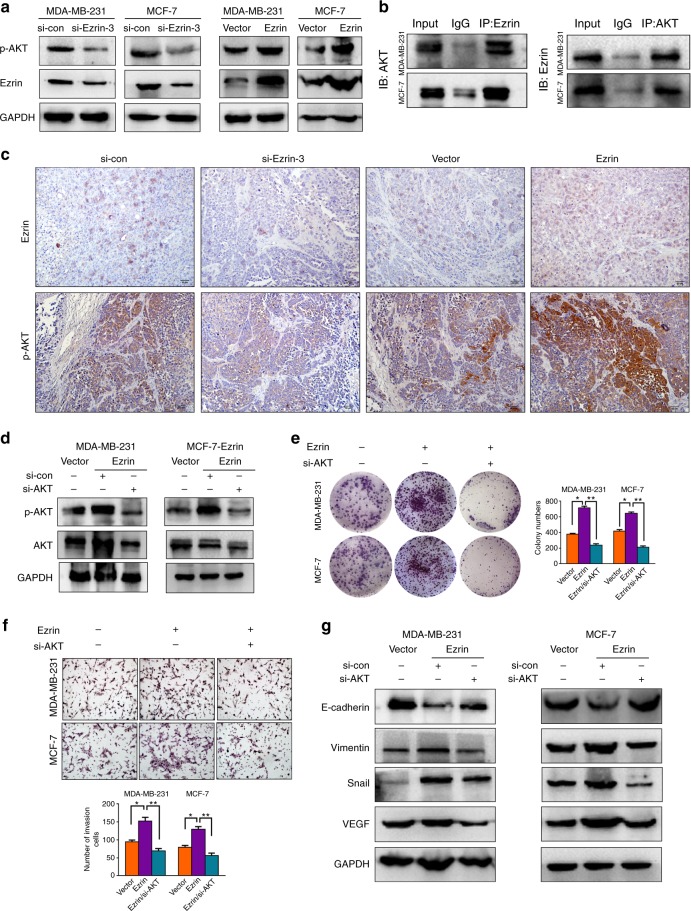
Ezrin interacts with AKT in BC. **a** The protein expression levels of p-AKT analysed by Western blot after knockdown or overexpression of Ezrin in BC cells. **b** Ezrin interacts with AKT. Lysates from MDA-MB-231 and MCF-7 cells were subjected to immunoprecipitation and western blot with the indicated antibodies. An irrelevant IgG was used as the IP control. **c** The correlation of Ezrin and p-AKT in the xenograft tumours tissues was detected by IHC. **d** AKT was knockdown in Ezrin overexpression cells. **e** Cell proliferation was examined by colony formation assay in constructed cells with or without AKT knockdown. **f** Cell invasion were investigated by transwell assays in constructed cells with or without AKT knockdown. **g** Western blot analysis of EMT markers and VEGF in constructed cells with or without AKT knockdown

Activation of AKT has frequently been reported in many human cancers, including carcinomas of the lung, pancreas and gastric, as well as in BC.^26^ In addition, this kinase appears to play an important role in cancer development, progression, and therapeutic resistance. We then investigated whether the activation of AKT is relevant to Ezrin expression in BC. Western blot analysis showed that the expression of p-AKT protein was significantly inhibited in BC cells with Ezrin knockdown but increased with Ezrin overexpression (Fig. 5a). We further performed co-immunoprecipitation in MDA-MB-231 and MCF-7 cells, our data revealed that Ezrin interacted with AKT in BC cells (Fig. 5b). Moreover, we detected the p-AKT expression in primary tumours formed by inoculation of BC cells transfected with the corresponding compounds in SCID mice by IHC. The results showed that p-AKT expression levels were commonly and positive correlated with Ezrin expression (Fig. 5c).

To further investigate whether Ezrin-mediated BC cells metastasis and angiogenesis are dependent on AKT activation, we knocked down AKT in Ezrin overexpressed cells by transfecting AKT siRNA (Fig. 5d). As shown in Fig. 5e, g, knockdown AKT can partially reverse the biological behaviours induced by Ezrin in BC cells. Taken together, these results demonstrated that AKT is required for Ezrin-mediated BC metastasis and angiogenesis.

### The tumour-promoting effects induced by Ezrin in BC are mediated through the activation of AKT signalling

**Fig. 6:**
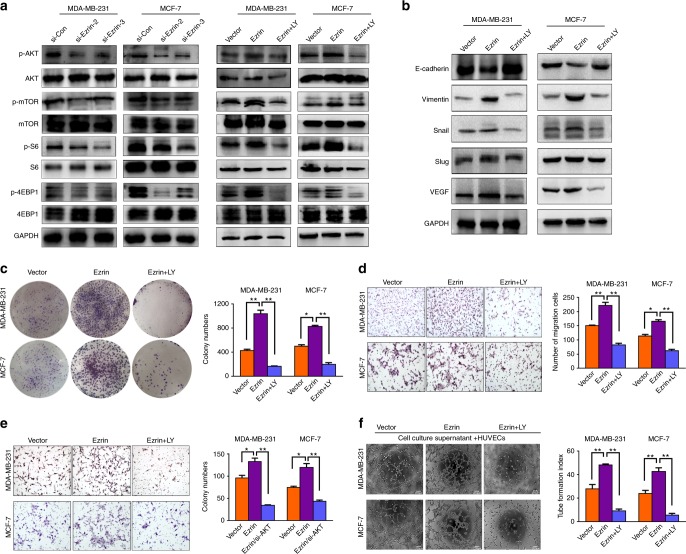
Ezrin overexpression promotes BC progression via AKT signalling pathway. **a** Western blot analysis of t-AKT/p-AKT, t-mTOR/p-mTOR, t-S6/p-S6 and t-4EBP1/p-4EBP1 in the constructed cells. GAPDH was used as the loading control. The protein expression levels of indicated genes were also analysed in Ezrin overexpressed cells treated with PI3K inhibitor LY294002 (25 μmol/l). **b** Western blot analysis of EMT markers and VEGF in constructed cells that had been treated with or without LY294002. **c** Colony formation assay was used to investigate the proliferation of constructed cells with or without LY294002. **d**, **e** Cell migration and invasion were evaluated by transwell assays in constructed cells with or without LY294002. **f** Vasculogenic mimicry assay were performed in constructed cells that had been treated with or without LY294002

Based on these findings, we hypothesised that Ezrin likely to induce metastasis and angiogenesis in BC cells via the AKT pathway. We assessed t-AKT/p-AKT, t-mTOR/p-mTOR, t-S6/p-S6, and t-4EBP1/p-4EBP1 expression levels by Western blot analysis. The results demonstrated that Ezrin-depleted cells had reduced, while Ezrin overexpressed cells had increased the levels of p-AKT, p-mTOR, p-S6 and p-4EBP1, with no significant change in the level of t-AKT, t-mTOR, t-S6 and t-4EBP1 (Fig. 6a). In line with this, we found that PI3K inhibitor LY294002 and mTOR inhibitor Rapamycin could markedly decreased the levels of p-AKT, p-mTOR, p-S6 and p-4EBP1 when compared with the Ezrin overexpressed group. Moreover, LY294002 and Rapamycin can also cause the down-regulation of Vimentin, Snail, Slug, and VEGF, as well as the up-regulation of E-cadherin in Ezrin overexpression BC cells (Fig. 6b) (Supplemental Fig. 4). In addition, we found that LY294002 and Rapamycin decrease the proliferation, migration, invasion and angiogenesis in Ezrin overexpressed cells (Fig. 6c-f) (Supplemental Fig. 4). In all, these results suggested that Ezrin promotes tumour progression at least in part via the AKT pathway.

## Discussion

Ezrin, which is the most important member of the ERM family, was primarily expressed in a variety of malignant tissues that originate from epithelial or non-epithelial cells.^27^ Wang et al. reported that Ezrin is positively related to lymph node metastasis of patients with TSCC, and high activities of Ezrin suggest poor prognosis of these patients,^28^ which suggested that Ezrin may function as an oncogene. However, Ezrin expression has also been reported to be down-regulated in human intrahepatic cholangiocarcinoma, and its loss was shown to result in a more aggressive phenotype. Therefore, these data indicated that Ezrin may also serve as a tumour suppressor.^29^ The discordance between these studies suggested that the effects of Ezrin in different tumours may be different.

To determine the potential functions of Ezrin in the pathogenesis of BC, we first assessed the expression of Ezrin in BC tissues by IHC, and found that the level of Ezrin was markedly higher in the BC tissues than that in the normal breast tissues. In addition to being used as a diagnostic marker for BC, we found that the high expression of Ezrin was associated with phenotypes of invasion and metastasis in BC, including tumour differentiation, late TNM stage and LN metastasis. Furthermore, patients with high Ezrin expression have a shorter survival time. A similar trend was also reported in pancreatic ductal adenocarcinoma and lung cancer,^30,31^ which supported our present findings. Thus, the expression of Ezrin can help predict the prognosis of patients with BC, and it may be a great tool for the proper management of personalised therapy.

An increasing amount of evidence indicate that Ezrin plays a central role in the development and progression of malignant tumours.^32,33^ Zhang et al. indicated that the overexpression of Ezrin affects the processes of hepatocellular carcinoma cell proliferation, migration, and invasion.^34^ These results are highly consistent with our previous studies in cervical cancer cells in which we reported the inhibitory effects of Ezrin knockdown on cell growth, migration, and invasion.^21^ In this study, we found that the proliferation, migratory and invasive abilities of BC cells in vitro were significantly inhibited with the depletion of Ezrin, and increased with the overexpression of Ezrin. We then extended our study to an in vivo xenograft model and demonstrated that Ezrin knockdown significantly reduced tumour growth and incidence of lung metastasis.

EMT, a well-characterised embryological process, contributes to enhance the ability of cancer cells to migrate and invade, which is critical in tumour metastasis. A recent study indicated that inhibiting the expression of Ezrin limited morphological changes and actin filament remodelling, thereby reducing the migration and invasion of cells during EMT.^35^ Here we demonstrated that when Ezrin was silenced, BC cells attained an epithelial morphology and lost their migratory capability. In addition, Ezrin knockdown clearly upregulated the expression of epithelial markers (E-cadherin and Zo-1), in agreement with the previous reports which uncovered that Ezrin depletion restored the membranous expression of E-cadherin and inhibited cytoplasmic expression of β-catenin in lung cancer cells.^31^ Additionally, we found that the levels of Vimentin and MMP9 were decreased following Ezrin depletion. Furthermore, transcriptional factors Snail and Slug were identified to be involved in Ezrin-regulated EMT. These results were further validated in an in vivo xenograft model, suggested that Ezrin can promote EMT during the progression of BC, thus exhibiting its pro-metastasis function.

Angiogenesis is necessary for continued tumour growth and is a prerequisite for metastasis. Inhibition of angiogenesis has been shown to prevent tumour progression and improve the outcomes in a range of tumour types.^36^ Ghaffari et al. reported that the expression of VEGF-A/-C and the activity of angio/lymphangiogenic signals were significantly reduced in Ezrin-deficient cells, thus showing a novel extrinsic mechanism by which Ezrin regulates the early stages of metastasis.^37^ In this study, we found that the expression of VEGF, which is the primary cytokine that promotes angiogenesis in solid tumours by inducing endothelial cell proliferation and migration and vascular permeability,^38^ were decreased following Ezrin depletion. In addition, our data demonstrated that Ezrin depletion disrupted tube formation in vitro and reduced pre-existing vasculature ex vivo. Furthermore, in vivo studies were further performed to evaluate the potential influence of Ezrin on angiogenesis. Together, our data, in accordance with previous reports, suggested that Ezrin has a crucial role in BC angiogenesis.

Numerous studies indicated that the deregulated of AKT can contribute to the development or progression of a wide variety of cancers. In addition, we^21^ and others^39^ have previously demonstrated that down-regulation of Ezrin can decrease the activity of AKT, while overexpression of Ezrin increase AKT activity. So there is considerable rationale for studying the relationship between Ezrin and AKT in BC. In the present study, we showed that Ezrin is required for AKT activation in BC cells, and knockdown of AKT reversed the effect of Ezrin overexpression in BC. Similarly, Krishnan et al. demonstrated that inhibition of Ezrin through expression of a non-phosphorylatable mutant results in reduction of phosphorylation of the serine/threonine kinase AKT at serine 473.^40^ Since then, we hypothesised that the influence of Ezrin in BC at least in part via the AKT pathway. As expected, the overexpression of Ezrin significantly induced EMT, migration and angiogenesis in BC. And the effects were suppressed by LY294002, the PI3K specific inhibitor, which can significantly reduce the phosphorylation of AKT. This finding was similarly to the one of Youn et al., they found that ezrin/calpain/PI3K/AMPK/AKT/eNOSs1179 pathway was critical for VEGF induction of endothelial nitric oxide (NO) production, which would, in turn, mediate VEGF-dependent angiogenesis.^41^ These findings broadened our understanding that Ezrin-regulated EMT via the AKT pathway, and suggested new strategies for the improvement of cancer therapeutics via the inhibition of AKT-mediated tumour metastasis.

Taken together, our observations suggested that Ezrin was upregulated in BC tissues compared with normal tissues, especially in metastatic BC. In addition, our study connects the interaction between Ezrin and AKT pathway to the promotion of BC cell behaviour associated with tumour progression (Supplemental Fig. 5). Thus, this study links Ezrin to BC progression and establishes Ezrin as a potential biomarker for predicting clinical prognosis and a therapeutic target in BC.

## Supplementary information

Supplemental Table

supplementary material

Supplemental Figure 5

## Data Availability

The data sets used and analysed during the current study are available from the corresponding author on reasonable request.
