# Supplementary material for: Ezrin promotes breast cancer progression by modulating AKT signals
Source: Br J Cancer. 2019 Feb 26;120(7):703–13. doi: 10.1038/s41416-019-0383-z (PMC6461860; doi:10.1038/s41416-019-0383-z)
Supplement: Supplementary file 1 — Supplemental Table [file 41416_2019_383_MOESM1_ESM.docx]

**Table 1. Relationship between Ezrin expression and clinicopathologic features of BC patients**

| **Clinical features** | **No. of cases** | **Strongly positive**  **cases (%)** | ***χ^2^*** | ***P* value** |
| --- | --- | --- | --- | --- |
| **Age**  ≥50  <50 | 63  54 | 38(60.3%)  36 (66.7%) | 0.504 | 0.478 |
| **Menopausal status**  Premenopausal  Postmenopausal | 45  72 | 27(60.0%)  47(65.3%) | 0.332 | 0.565 |
| **Tumor size**  <3.0  ≥3.0 | 61  56 | 39 (63.9%)  35 (62.5%) | 0.026 | 0.872 |
| **Tumor differentiation**  Well  Moderate  Poor | 48  51  18 | 23(47.9%)  37(72.5%)  14(77.8%) | 8.387 | 0.015* |
| **Clinical stage**  0-II  III-IV | 64  53 | 33 (51.6%)  41 (77.4%) | 8.299 | 0.004** |
| **LN metastasis**  Negative  Positive | 66  51 | 34 (51.5%)  40 (78.4%) | 8.967 | 0.003** |

* *P*<0.05 and ** *P*<0.01
