# Supplementary material for: Ezrin promotes breast cancer progression by modulating AKT signals
Source: Br J Cancer. 2019 Feb 26;120(7):703–13. doi: 10.1038/s41416-019-0383-z (PMC6461860; doi:10.1038/s41416-019-0383-z)
Supplement: Supplementary file 2 — supplementary material [file 41416_2019_383_MOESM2_ESM.doc]

**Supplementary Figure Legends**

**Supplemental Fig.1:** Western blot analyses of Ezrin expression in MDA-MB-231 and MCF-7 cells transiently transfected with Ezrin-targeting siRNA (si#1, si#2 and si#3).

**Supplemental Fig.2: Ezrin promotes EMT in BC cells.** (A) The morphology of the constructed MDA-MB-231 and MCF-7 cells. (C) The protein expression levels of EMT markers in the indicated cells were detected by IF. DAPI (blue) was used to mark the nuclei.

**Supplemental Fig.3: Ezrin depletion inhibits tumor angiogenesis.** (A) Western blot analysis of Ezrin and p-Ezrin of HUVECs in different culture conditions. (B) Tube formation assay was performed in HUVECs incubated in culture supernatant from HUVECs cells 48 h after Ezrin depletion or not.

**Supplemental Fig.4: Ezrin overexpression promotes BC progression via AKT signaling pathway.** (A) Western blot analysis of t-mTOR/p-mTOR, t-S6/p-S6 and t-4EBP1/p-4EBP1 in the constructed cells. GAPDH was used as the loading control. The protein expression levels of indicated genes were also analyzed in Ezrin overexpressed cells treated with mTOR inhibitor rapamycin. (B) Western blot analysis of EMT markers and VEGF in the constructed cells that had been treated with or without rapamycin. (C) Colony formation assay was used to investigate the proliferation of constructed cells with or without rapamycin. (D) Cell invasion were evaluated by transwell assays in the constructed cells with or without rapamycin. (E) Vasculogenic mimicry assay were performed in the constructed cells treated with or without rapamycin.

**Supplemental Fig.5: Schematic diagram.**
