# Supplementary figures and images for: Ezrin promotes breast cancer progression by modulating AKT signals
Source: Br J Cancer. 2019 Feb 26;120(7):703–13. doi: 10.1038/s41416-019-0383-z (PMC6461860; doi:10.1038/s41416-019-0383-z)

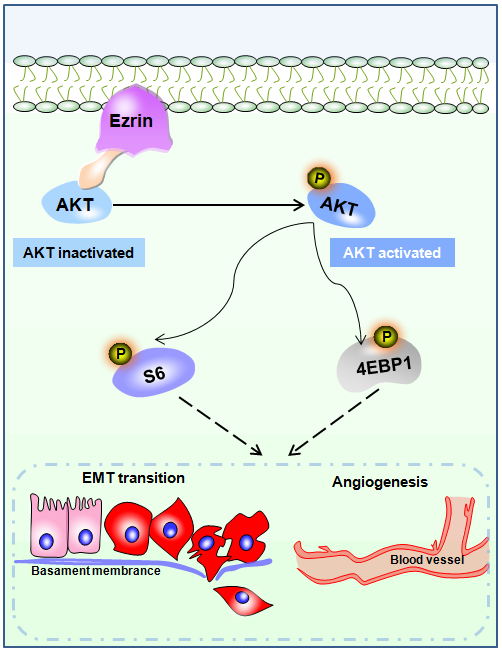

Supplement: Supplementary file 3 — Supplemental Figure 5 [file 41416_2019_383_MOESM3_ESM.tif]
